# Supplementary material for: Enhancing knowledge of hypertension among general practitioners in Pakistan through a Train-The-Trainer initiative
Source: PLoS One. 2025 Aug 11;20(8):e0328447. doi: 10.1371/journal.pone.0328447 (PMC12338800; doi:10.1371/journal.pone.0328447)
Supplement: Attachment — Submitted filename: Response to Reviewers.docx [file pone.0328447.s002.docx]

**Dear Editor,**

Thank you for sharing valuable feedback of reviewers regarding our submission to the PLOS ONE titled **“Enhancing Hypertension Knowledge among General Practitioners in Pakistan through Train the Trainer Initiative”**. Manuscript ID: [PONE-D-24-59868] - [EMID:e69cd29e62458246]

We have revised the manuscript as per the specific comments as listed below. We believe these changes have strengthened the quality of our manuscript and that you will find it suitable for publication in the Journal.

**Journal requirements:**

**Comment:** When submitting your revision, we need you to address these additional requirements.

1. Please ensure that your manuscript meets PLOS ONE's style requirements, including those for file naming. The PLOS ONE style templates can be found at

https://journals.plos.org/plosone/s/file?id=wjVg/PLOSOne_formatting_sample_main_body.pdf and

https://journals.plos.org/plosone/s/file?id=ba62/PLOSOne_formatting_sample_title_authors_affiliations.pdf

**Response:** Title page and manuscript formatting is updated as per the PLOS ONE's style requirements

**Comment:** 2. PLOS requires an ORCID iD for the corresponding author in Editorial Manager on papers submitted after December 6th, 2016. Please ensure that you have an ORCID iD and that it is validated in Editorial Manager. To do this, go to ‘Update my Information’ (in the upper left-hand corner of the main menu), and click on the Fetch/Validate link next to the ORCID field. This will take you to the ORCID site and allow you to create a new iD or authenticate a pre-existing iD in Editorial Manager.

**Response:** The ORCID iD for the corresponding author is now linked in the editorial manager

**Comment:** 3. Your ethics statement should only appear in the Methods section of your manuscript. If your ethics statement is written in any section besides the Methods, please delete it from any other section.

**Response:** Ethics statement is removed from elsewhere

**Comment:** 4. We note that there is identifying data in the Supporting Information file < S1_File.xlsx>. Due to the inclusion of these potentially identifying data, we have removed this file from your file inventory. Prior to sharing human research participant data, authors should consult with an ethics committee to ensure data are shared in accordance with participant consent and all applicable local laws.

Data sharing should never compromise participant privacy. It is therefore not appropriate to publicly share personally identifiable data on human research participants. The following are examples of data that should not be shared:

-Name, initials, physical address

-Ages more specific than whole numbers

-Internet protocol (IP) address

-Specific dates (birth dates, death dates, examination dates, etc.)

-Contact information such as phone number or email address

-Location data

-ID numbers that seem specific (long numbers, include initials, titled “Hospital ID”) rather than random (small numbers in numerical order)

Data that are not directly identifying may also be inappropriate to share, as in combination they can become identifying. For example, data collected from a small group of participants, vulnerable populations, or private groups should not be shared if they involve indirect identifiers (such as sex, ethnicity, location, etc.) that may risk the identification of study participants.

Additional guidance on preparing raw data for publication can be found in our Data Policy (https://journals.plos.org/plosone/s/data-availability#loc-human-research-participant-data-and-other-sensitive-data) and in the following article: http://www.bmj.com/content/340/bmj.c181.long.

Please remove or anonymize all personal information, ensure that the data shared are in accordance with participant consent, and re-upload a fully anonymized data set. Please note that spreadsheet columns with personal information must be removed and not hidden as all hidden columns will appear in the published file.

**Response:** The data file is reviewed and efforts are made to omit any identity information

**Additional Editor Comments:**

**Comment:** Please ensure that the manuscript adheres to the PLOS One formatting guidelines, particularly in terms of using subheadings in the abstract and appropriately placing tables and figures as specified in the guidelines. The "Clinical Perspective" section, located after the conclusion, does not follow the PLOS One formatting structure. It would be better to incorporate this information as a summary elsewhere, possibly within the discussion section.

**Response:** The abstract is formatted in according with PLOS One formatting structure and the "Clinical Perspective" section has been omitted.

**Comment:** Furthermore, there is substantial room for improvement in the discussion section. Instead of merely reiterating the results, please include more argumentative points from the authors, providing appropriate comparisons and highlighting the future implications of the findings.

**Response:** As per the suggestions, the discussions were elaborated where ever needed.

**5. Review Comments to the Author**

Reviewer #1: The manuscript is suitable for PLOS ONE, as it aligns with the journal's scope of publishing scientifically rigorous research without a focus on novelty. However, to improve its chances of acceptance, the authors should consider the following revisions:

Major Revisions:

**Comment:** Clarity & Conciseness:

The manuscript is overly wordy in several sections, especially in the Introduction and Discussion. Reducing redundancy and improving clarity will enhance readability.

Example: The background on hypertension prevalence and management in LMICs can be streamlined to focus more on Pakistan-specific challenges.

**Response:** Thank you for the valuable feedback, in accordance with the suggestions we have modified introduction and discussion section where ever needed.

**Comment:** Study Design & Methodology:

The manuscript states a "quasi-experimental design," but does not specify the control group or if there was a comparison with a non-trained group. Clarify this aspect.

Explain how GPs were recruited for training—was it voluntary, randomized, or based on a specific criterion?

**Response:** Thank you for the valuable feedback, there was not an explicit control group, but the pre-training knowledge level of the participants with the 1-3 months post knowledge level of same participants. Participants were selected based on non-probability convenient sampling methods as mentioned in the methods section.

**Comment:** Data Analysis & Interpretation:

The statistical methods are generally appropriate but require more details. Report effect sizes or confidence intervals to strengthen claims of improvement.

While p-values are provided, consider discussing the clinical relevance of observed improvements.

**Response:** Thank you for the suggestions, we have modified the content

**Comment:** Limitations Section:

The limitations should explicitly mention the potential biases due to self-selection and self-reported assessments.

Discuss the lack of long-term follow-up—while knowledge improved, was there any evidence of sustained application in clinical practice?

**Response:** Thank you for the suggestions, we have modified limitation section

**Comment:** Figures & Tables:

The manuscript references multiple figures and tables, but their descriptions could be more precise. Ensure that each figure clearly supports the findings without unnecessary redundancy.

**Response:** Thank you for the suggestions, we attempted to modify details of figures and table wherever necessary but redundancy is avoided.

Minor Revisions:

Grammar & Formatting:

**Comment:** Several grammatical errors and awkward phrasings need revision. Example: "Study participants were consisted of 540 general practitioner working across Pakistan" should be "The study included 540 general practitioners working across Pakistan."

**Response:** Thank you for the suggestions, we have reviewed content for grammatical errors.

**Comment:** Ensure consistency in terminology—sometimes "GPs" is used, other times "general practitioners."

**Response:** Thank you for the suggestions, we have updated terminology.

Ethics & Funding Clarifications:

**Comment:** The ethics statement mentions approval from the Pakistan Medical Association, but ensure it meets PLOS ONE's guidelines (e.g., include participant consent procedures clearly).

The funding statement currently states “sponsored by PharmEvo Private Limited.” Clearly outline if the sponsor had any role in study design, execution, or manuscript preparation.

**Response:** Thank you for the valuable feedback, consent details and sponsor role are updated.

**Reviewer #2: Dear Authors,**

I read with deep interest your manuscript on the Train The Trainer (TTT) Initiative to Enhance Hypertension Initiatives in Pakistan. I really found the manuscript interesting and insightful, and believe the topic is of significant public health importance.

I do wish to make some suggestions aimed solely at augmenting the quality of the manuscript.

Please find below:

**Comment:** 1. Introduction:

While the discussion on the public health importance of hypertension and its economic impact is comprehensive, incorporating a brief exploration of general practitioners’ perspectives—such as their confidence in diagnosing and managing hypertension—could further enrich this section. Including additional literature from Pakistan, for instance, the manuscript by Farazdaq et al. (see link below), may help underscore the existing gaps in clinical knowledge and the willingness of GPs to enhance their skills through initiatives like the TTT program.

https://journals.lww.com/jfmpc/fulltext/2022/12000/Needs_assessment_of_general_practitioners_in.18.aspx

**Response:** Thank you for the valuable feedback, we have incorporated the suggested study in the introduction section

**Comment:** 2. Statistical Analysis:

May the authors elaborate further on which statistical software or tool (e.g. Epidata) was used to calculate the sample size?

**Response:** Thank you for the valuable feedback, we have added software details for sample size calculation

3. Data Security:

**Comment:** Given the online and electronic nature of the survey instrument, what specific measures did the authors take to ensure all GPs who participated in the survey's data remained secure? A brief section outlining data management and security measures in the manuscript will be very helpful.

**Response:** Thank you for the valuable feedback, we have added details regarding security measures.

**Comment:** 4. Discussion:

The studies cited regarding the prevalence of hypertension in Pakistan are over seven years old, hence it would be helpful if the authors could consider referencing the latest Global Burden of Disease (GBD) study on hypertension, which provides updated statistics relevant to Pakistan. The link is below:

https://www.thelancet.com/journals/eclinm/article/PIIS2589-5370(23)00211-0/fulltext

**Response:** Thank you for the valuable feedback, we have incorporated the suggested study in the discussion section

**Comment:** 5. Funding:

Was there a specific grant provided by PharmEvo for the training session? If so, can a specific grant reference number be provided and mentioned in the Funding section of the manuscript?

**Response:** Thank you for the suggestions, this this was not a format grant hence no grant reference number to provide.

**Comment:** 6. Survey Completion:

Were there any incomplete online survey forms filled by the general practitioners for the study? And if so, how did the authors treat the incomplete online survey forms in their analysis?

**Response:** Thank you for the suggestions, we have made all the questions mandatory on the survey form, hence there were no missing response, however, the contact could not be established for follow-up assessment with 7 GPs who were excluded from the final analysis. This has been mentioned in the analysis section.

**Comment:** 7. Instrument Validation:

Since the authors used a modified version of the instrument by Chen Q et al edited for local contexts, did they do a pilot to ensure accurate administration and distribution of the instrument and it's suitability for local contexts? And did they include any results in the final analysis?

**Response:** Thank you for the valuable feedback, yes the modified questionnaire was tested in a pilot phase of 30 respondents, details are added to the methods seciotn.

**Comment:** 8. Language and Grammar:

There are some minor grammatical errors in the sentence structure of the manuscript. For example, the phrase “training of 33 volunteer consultant cardiologist” should be revised to “training of 33 volunteer consultant cardiologists.” in line 150 of the manuscript. A second proofread by a native English speaker, especially of the Methodology section, will help correct these minor lapses.

**Response:** Thank you for the suggestions, we have reviewed content for grammatical errors.
