# Supplementary material for: Enhancing knowledge of hypertension among general practitioners in Pakistan through a Train-The-Trainer initiative
Source: PLoS One. 2025 Aug 11;20(8):e0328447. doi: 10.1371/journal.pone.0328447 (PMC12338800; doi:10.1371/journal.pone.0328447)
Supplement: Attachment — Submitted filename: Response_to_Reviewers_auresp_2.docx [file pone.0328447.s003.docx]

**Dear Editor,**

Thank you for sharing valuable feedback of reviewers regarding our submission to the PLOS ONE titled **“Enhancing Hypertension Knowledge among General Practitioners in Pakistan through Train the Trainer Initiative”**. Manuscript ID: [PONE-D-24-59868R1] - [EMID:6454c729025537b3]

We have revised the manuscript as per the specific comments as listed below. We believe these changes have strengthened the quality of our manuscript and that you will find it suitable for publication in the Journal.

**Review Comments to the Author**

Overall, the work is of good quality; however, several issues—particularly in the Discussion section—still require your attention. Please consider the following detailed suggestions:

**Comment 1.** Ethics Statement and Financial Disclosure

--> There is an inconsistency between the manuscript and the information submitted in the portal regarding the Ethics Statement and Financial Disclosure (specifically regarding funding from PharmEvo Private Limited).

--> Although the manuscript has been updated per the reviewer’s suggestion in the Methods section and the Declarations section, the corresponding section on the journal’s submission portal under Financial Disclosure and Ethics Statement in the Additional Information section must also be updated accordingly by the submitting author to ensure consistency throughout the submission.

**Response:** Thank you for your comments, we have modified Ethics Statement and Financial Disclosure on the portal.

**Comment 2**. Availability of Data and Materials

--> The manuscript states in the Declarations section that data will be available upon reasonable request, whereas the Data Availability Statement in the section under Additional Information in the journal submission portal indicates that the data are fully available without restriction as a supplementary file. Please reconcile these differences to ensure consistency between the two sources.

**Response:** Thank you for your comments, we have modified data availability statement on the portal.

3. Introduction Section

While the Introduction provides a thorough discussion of hypertension, it currently suffers from excessive length and redundancy:

**Comment** --> Approximately two paragraphs are dedicated to discussing the prevalence of hypertension, its economic burden, and the consequences of uncontrolled hypertension. Although these details are important, they may overwhelm busy general physicians and PLOS One readership who are not necessarily specialists in the field and hence, should be trimmed down.

--> The Introduction should briefly review existing literature—particularly on Train-the-Trainer initiatives for hypertension management globally—highlight the gaps in the literature, and clearly state how your study addresses these gaps (if any).

--> The final paragraph appears abruptly without a clear connection to the preceding content, if a brief review of the current literature on the topic especially on Train-The-Trainer initiatives for hypertension is included, it will flow naturally to this portion of the introduction.

Suggestion: Trim the Introduction to no more than five paragraphs, ensuring that the literature gaps are explicitly discussed.

**Response:** Thank you for pointing it out, we have trimmed down the introduction as per the suggestions.

4. Discussion Section

The Discussion section also requires revision for clarity and coherence. Please consider the following specific points:

a) Citation Needed:

--> In lines 226-227, please provide a citation for the statement: “Hypertension is the medical condition defined by chronic elevation of arterial pressure in the systemic arteries above threshold values that have been established.”

**Response:** Thank you for pointing it out, we have added the citation.

b) Statistical Correction and Reference Update:

--> The sentence “For instance, one study estimated the rate as 19.1% from 1990 to 1994 [20] using data from the National Health Survey” in line 237 needs two corrections. First, the paper by Tazeen Jafer et al. actually reports the rate as 19.0%. Second, the reference is incorrectly cited as [20]; it should be citation [21] in the reference list. Please amend both aspects accordingly.

**Response:** Thank you for pointing it out, we have rectified both typographic errors

c) Citation Placement:

--> In the sentence “A couple of years ago, a more recent national health survey conducted in 2010 estimated that 33% of adults aged 45 years and 18% of all adults in Pakistan were hypertensive,” please place the citation for Saleem F et al. (citation [24]) at the beginning of the sentence.

**Response:** Thank you for pointing it out, we have added the citation.

d) Flow and Transition:

--> The statement referencing the Global Burden of Disease (GBD) study is inserted abruptly in lines 242-244. Since the following paragraph discusses Saleem F et al. (citation 24), please revise the transition between these two sections to ensure a smoother flow.

**Response:** Thank you for pointing it out, we have modified the flow of paragraphs.

e) Abbreviation Introduction:

--> The abbreviations ABPM (line 289), HBPM, and OBPM (line 315) are introduced without first providing their full forms. Given the diverse readership of PLOS ONE, please ensure that the full form of each abbreviation is provided at its first occurrence in the manuscript.

**Response:** Thank you for pointing it out, we have added full forms of the abbreviations

f) Structural Revision:

As noted by another reviewer and the editor, the discussion is quite lengthy and meandering. A more focused structure would:

--> Start by directly addressing each key finding of the study.

--> Compare and contrast these findings with similar studies in the literature.

**Response:** Thank you for the suggestion, we have moved background information to the introduction section and discussion is now starts with a small background followed by the main findings of the study.

--> Whilst lines 324- 338, highlight the implications of the findings in accordance with the editor's suggestions, it is important, at the same time, to insert appropriate citations with the statements of fact specifically in these two paragraphs.

An example of this would be to provide a citation for the statement "targeted training among GPs, as they are usually the first contact for the hypertensive patient.", and other similar statements in these two paragraphs, which are not inferential in nature but are statements of fact or claim and hence need the appropriate citations from the literature.

**Response:** Thank you for pointing it out, we have added the citation.

--> Consider moving the initial two paragraphs—which primarily discuss the clinical definition of hypertension and the epidemiology of hypertension in Pakistan—to the Introduction. Use the Discussion to delve deeply into your study’s findings and their implications relative to previous similar research. This restructuring should help maintain the reader’s attention while providing a concise analysis.

**Response:** Thank you for the suggestion, we have moved background information to the introduction section as per the suggestions.

5. Manuscript Title

--> Please consider updating the main title to: "Enhancing Knowledge of Hypertension among General Practitioners in Pakistan through a Train-The-Trainer Initiative." This is more grammatically accurate.

**Response:** Thank you for the suggestion, the title is modified as per the suggestions.

--> Please also ensure that this title is consistently used throughout the manuscript as well as the submission in the portal and that the capitalization of the words is appropriate.

**Response:** Thank you for the suggestion, the title is modified in the submission portal too.

6. Grammar Check

--> It is also suggested that the authors proof read the Discussion and conclusion section of the manuscript as there are still some minor lapses (such as in 353 where the words 'might affect' should be replaced by the words 'might result in') in grammar.

**Response:** Thank you for the suggestion, the term is modified as per the suggestions.

--> Either proofreading the entire manuscript one more time by a fluent English speaker or using software like Grammarly may help correct these minor grammatical lapses throughout the manuscript.

**Response:** Thank you for the suggestion, the manuscript has been reviewed for grammatical corrections.
