# Supplementary material for: Enhancing knowledge of hypertension among general practitioners in Pakistan through a Train-The-Trainer initiative
Source: PLoS One. 2025 Aug 11;20(8):e0328447. doi: 10.1371/journal.pone.0328447 (PMC12338800; doi:10.1371/journal.pone.0328447)
Supplement: Attachment — Submitted filename: Response_to_Reviewers_auresp_3.docx [file pone.0328447.s004.docx]

**Dear Editor,**

Thank you for sharing valuable feedback of reviewers regarding our submission to the PLOS ONE titled **“Enhancing Knowledge of Hypertension among General Practitioners in Pakistan through a Train-The-Trainer Initiative”**. Manuscript ID: [PONE-D-24-59868R2] - [EMID:aebbf8c8c24dc0d2]

We have revised the manuscript as per the specific comments as listed below. Thank you for sharing valuable feedback.

**Journal Requirements:**

Please review your reference list to ensure that it is complete and correct. If you have cited papers that have been retracted, please include the rationale for doing so in the manuscript text, or remove these references and replace them with relevant current references. Any changes to the reference list should be mentioned in the rebuttal letter that accompanies your revised manuscript. If you need to cite a retracted article, indicate the article’s retracted status in the References list and also include a citation and full reference for the retraction notice.
**Response**: Thank you for the comments, we have rechecked all the references for the completeness.

**Additional Editor Comments:**

Please address reviewers minor comments

**Reviewer #2:**

Dear Authors, I read with deep interest your revised manuscript. Thank You for incorporating the suggestions in the paper. I find the work to be fantastic and of significant public health importance. I think the manuscript can now be accepted in its current status. I just had 2 queries which can be addressed in the final proof version upon acceptance by the authors:

**Comment 1.** Were any Research Associates hired to enroll the GPs for this study? Or for any relevant particular purpose of the study? If so, please do acknowledge them with names for this national initiative level study in the final proof version of the manuscript.

**Response**: Thank you for the feedback. We have included the acknowledgment for the research officers as suggested.

**Comment 2.** Whilst inclusion criteria are in the study, was there any exclusion criteria for the GPs in the study? If so, it can be specified in the final proof version of the manuscript. As the authors did exclude GPs of a certain criteria in a past work of theirs: <https://www.sciencedirect.com/science/article/pii/S2772628224000335>

**Response**: Thank you for your comment, to minimize potential bias and confounding effects from recent training, GPs who had attended a structured HTN education or training program within the past six months were excluded from the study. Same has been added to the methods section.
